# 

**Published:** 1962

**Authors:** 


### THE MEDICAL JOURNAL OF THE SOUTH-WEST

*Incorporating*
*The Bristol Medico-Chirurgical Journal*

NATIONAL LIBRARY
MAR 8 1963
OF MEDICINE

*June/October 1962*

VOL. 77 (iii–iv). No. 285/286

